# Characteristics of the Motor Units during Sternocleidomastoid Isometric Flexion among Patients with Mechanical Neck Disorder and Asymptomatic Individuals

**DOI:** 10.1371/journal.pone.0167737

**Published:** 2016-12-12

**Authors:** Chia-Chi Yang, Fong-Chin Su, Po-Ching Yang, Hwai-Ting Lin, Lan-Yuen Guo

**Affiliations:** 1 Department of Biomedical Engineering, National Cheng Kung University, Tainan, Taiwan; 2 Medical Device Innovation Center, National Cheng Kung University, Tainan, Taiwan; 3 Department of Sports Medicine, College of Medicine, Kaohsiung Medical University, Kaohsiung, Taiwan; Mayo Clinic Minnesota, UNITED STATES

## Abstract

Mechanical neck disorder is a widespread and non-neurological musculoskeletal condition resulting from modern lifestyles. Presently, the fundamental electrophysiological properties of the motor units of the sternocleidomastoid muscles and the characteristics of the short-term synchronization of the motor unit in patients with neck pain are ambiguous. This study therefore aims to clarify the fundamental electrophysiological properties of the motor units of the sternocleidomastoid muscles in patients with mechanical neck disorder and in asymptomatic individuals. We further investigated whether alterations in the degree of motor unit short-term synchronization occur. The surface electrophysiological signals of the bilateral sternal heads of the sternocleidomastoid muscles of twelve patients with mechanical neck disorder and asymptomatic individuals were detected at 25% of the maximum voluntary contraction during cervical isometric flexion and then decomposed into individual motor unit action potential trains. We found that the patients with mechanical neck disorder showed significantly higher initial and mean firing rates of the sternocleidomastoid muscles and displayed substantially lower motor unit short-term synchronization values compared with the asymptomatic subjects. Consequently, these convincing findings support the assertion that patients with mechanical neck disorder display altered neuromuscular control strategies, such as the reinforcement of motor unit recruitment firing rates in the sternocleidomastoid muscles. The motor units of these patients also revealed neural recruitment strategies with relatively poor efficiency when executing the required motor tasks.

## Introduction

Mechanical neck disorder (MND) is commonly considered a widespread and unnerved musculoskeletal condition. Its threats to general health are becoming increasingly severe as technology advances in many industries. MND is defined as generalized neck pain and disability without any abnormal anatomical structures or identifiable orthopedic and neurological problems, such as spinal vertebral fracture, exceptional spinal lordosis, spinal spondylosis, spinal osteoarthritis and so on [1]. Clinically, the symptoms of neck pain, abnormal cervical kinematics, neck muscle weakness and neck-related functional disabilities are the common complaints for patients with MND [1–10]. Although the etiology of MND is largely under debate, it is believed that prolonged workloads and poor work postures are potential underlying causes [11–13]. Furthermore, psychosocial risk factors also have been reported as potential contributors [14].

A number of studies have investigated the association between neck pain and the neuromuscular control strategies of neck muscles. Generally speaking, neck pain is associated with increased activation and greater fatigability of the superficial cervical flexor muscles and the upper trapezius [15, 16] and reduced activation of the deep cervical flexor muscles [17]. Therefore, the hypothesis that increased activation of the superficial cervical flexor muscles, such as the sternocleidomastoid muscles, is a compensatory adaption for remedying the abnormal activation of the deep cervical flexor muscles has been widely accepted.

Because motor units are the fundamental functional units of the neuromuscular systems, the recruitment and derecruitment of the motor units or the pattern of the discharge rates of activated motor units have been proven to manipulate the forced production of the neuromuscular systems [18]. Additionally, observation of the electrophysiological properties of motor units could further determine the potential pathogenic mechanisms underlying various neuromuscular disorders [19–30]. For instance, Kallenberg et al. investigated differences in the properties of the motor units of the upper trapezius in patients with chronic neck-shoulder pain, compared to healthy controls and found significantly increased root-mean-square values of the motor unit action potentials and elevated median power frequencies during work-related tasks among those with chronic neck-shoulder pain [22].

Along with these basic myoelectric properties, the motor unit short-term synchronization describes an above-chance tendency for near-simultaneous discharges between pairs of motor units. It is believed that the phenomenon of motor unit synchronization is a deliberate neuromuscular strategy originated from shared synaptic inputs from the branching of last-order neurons [31, 32] and plays a pivotal role in regulating the mechanical output of neuromuscular systems [33–35]. The physiological modulations of motor unit synchronization not only imply the efficient means of achieving higher force or maintaining force during constant contraction but also coordinate the multiple muscular activations in synergy. Previous works have discussed the functional significance of motor unit synchronization in neuromuscular systems and has verified disruptions in motor unit synchronization in motor-related pathological conditions, such as sporadic amyotrophic lateral sclerosis and Klippel-Feil syndrome [36–37]. Also, reductions in motor unit synchronization are exhibited in patients with cerebral palsy and stroke [38–41].

However, investigations exploring the fundamental electrophysiological properties of the motor units of the sternocleidomastoid muscles in patients with MND are still rare. Additionally, no prior reports have described the features of the motor unit short-term synchronization of the sternocleidomastoid muscles. Therefore, the objectives of the current work are to clarify the fundamental electrophysiological properties of the motor units of the sternocleidomastoid muscles and to determine whether differences in the degree of motor unit short-term synchronization exist among patients with MND and asymptomatic individuals during cervical isometric flexion.

## Materials and Methods

### Participants

Two groups of volunteers were recruited to participate in the present investigation. Twelve patients with physician-diagnosed MND (2 males & 10 females, 28.8±8.1 years) who had sought medical treatment within the past 6 weeks participated voluntarily in the present study. The second group consisted of twelve asymptomatic individuals (5 males & 7 females, 27.5±6.2 years). None had any history of cervical surgery, cervical trauma, cervical pain or neuromuscular problems. Additional demographic details were initially recorded for each participant and are summarized in Table 1. Moreover, the level of functional disability of the neck was assessed using the Neck Disability Index for all patients with MND [42].

**Table 1:** Characteristics of the demographic data for patients with MND and asymptomatic individuals.

	Patients with MND (n = 12)	Asymptomatic Individuals (n = 12)	*P*-value
Age (years)	28.8 (8.1)	27.5 (6.2)	0.713
Gender	2 males, 10 females	5 males, 7 females	0.185^‡^
Height (cm)	161.5 (6.2)	169.8 (8.0)	0.012
Weight (kg)	55.3 (8.1)	64.3 (11.4)	0.101
NDI score*	15.8 (5.4)^†^		

*NDI: Neck disability index

^†^Mann-Whitney *U* test was used to identify the differences in age, height and weight

^‡^Chi-square test was used to verify gender proportion between healthy and MND groups

### Ethics Statement

All participants clearly understood the major aims of the study and signed consent forms prior to participating in the investigations. All the experimental procedures of the present investigation were approved by the Institutional Review Board of Kaohsiung Medical University Chung-Ho Memorial Hospital (No. KMUH-IRB-20120078).

### Apparatus for electromyography signal detection

The surface electrophysiological signals were detected from the sternal head of the sternocleidomastoid muscles bilaterally using the non-invasive dEMG^TM^ system (Delsys, Inc., Boston, Massachusetts). The accuracy of this system has been certified to be 92.5% on average. The dEMG^TM^ sensor consists of five pin electrodes; four of five electrodes are arranged in a 5-mm square, and the fifth pin electrode is located in the center of the square. This arrangement enables four channels of surface differential electrophysiological signals to be recorded simultaneously. To measure voluntary isometric contraction force during cervical flexion, a custom-designed force-measuring device with a tension/compression minibean load cell was made and anchored to an examination couch.

### Experimental procedures

**Fig 1:**
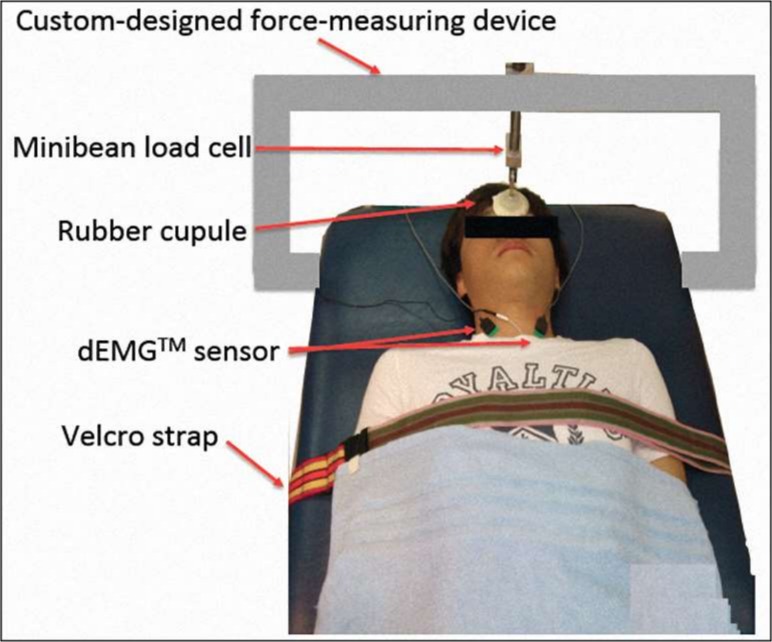
EMG acquisition set-up. Experimental set-up for collecting the EMG signals of the bilateral sternocleidomastoid muscles.

Because there are no obvious dominance effects of myoelectric activation between the bilateral sternocleidomastoid muscles in normal populations and because the prevalent pain locations were on the bilateral upper trapezius muscles in all the patients with MND in this investigation, the surface electrophysiological signals were detected from the sternocleidomastoid muscles bilaterally and pooled for further analysis. After familiarization with all experimental protocol, each participant was comfortably positioned in a supine position on an examination couch with arms positioned on the each side of the trunk and the trunk fastened via an adjustable strap to avoid unnecessary movements. Each participant was instructed to position the head in at midline, with an imaginary line extending from the forehead to the nose-bridge and running parallel to the load cell. Subsequently, the skin over the bilateral sternocleidomastoid muscles was cleansed using an alcohol swab. The dEMG^TM^ sensors were then placed over the belly of the sternal head of the sternocleidomastoid muscles bilaterally and fixed with adhesive tape. The ground electrode was attached over the sternal end of the clavicle, and a load cell was affixed to the custom-designed force-measuring device, which was attached firmly to the forehead of each participant (Fig 1).

After the experimental set-up was completed and the participant had practiced the required movements several times, each participant was asked to perform three brief maximum voluntary isometric contractions lasting three seconds. An interval of at least five minutes was provided between each repetition. The highest value of maximum voluntary isometric contraction force among these three repetitions was selected as the reference MVC and used to normalize the force level for further comparison. After the determination of the reference MVC, each participant was asked to follow a serious of trajectories of the target force displayed on a visual feedback monitor for each measurement. The trajectories of the target force followed a trapezoidal paradigm, which increased at a rate of 10% MVC/s, then sustained sub-maximal cervical isometric contraction at 25% MVC for 20 seconds, and finally decreased at a rate of 10% MVC/s. A rest period of at least ten minutes was provided between each measurement to avoid fatigue effects. Verbal encouragement was also provided to motivate each participant to execute cervical flexion contraction as close as possible to the target force. The surface analog electrophysiological signals from four differential channels were band-pass filtered at 20 Hz (12 dB/octave) to 1750 Hz (24 dB/octave), digitized with 16-bit resolution at a sampling rate of 20 kHz and stored on a personal computer for decomposition processing. In addition, the raw EMG signals had to successfully pass the certification of the Delsys Signal Quality Check, with the signal to noise ratio of > 2, baseline noise < 4.8 μV root mean square, and line interference < 1.0 to be acceptable for decomposition processing, according to the user’s guide. Next, the Precision Decomposition III algorithm was utilized to decompose the resulting digital electrophysiological signals into their constituent motor unit action potential trains [43, 44].

### Data processing

**Fig 2:**
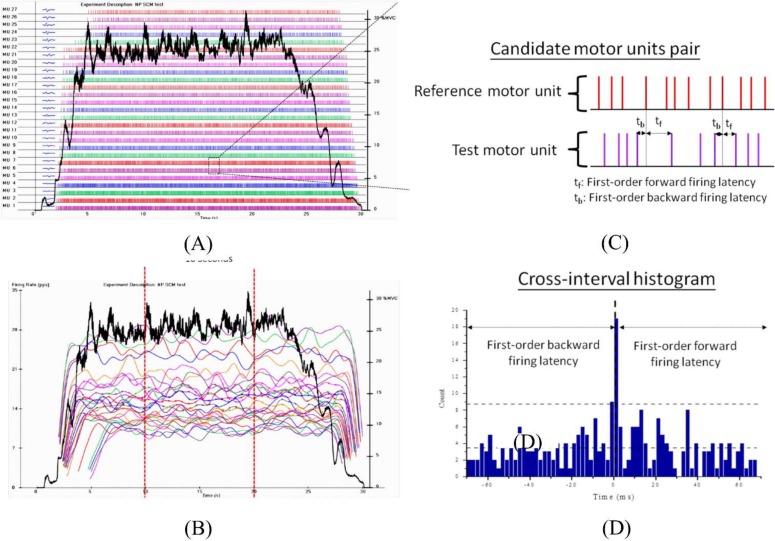
Representations of the firing properties of identified motor unit and the constructed cross-interval histogram of the test motor unit. (A, B) Illustrations for the firing instances and mean firing rates of the 27 identified motor unit of the unilateral sternocleidomastoid muscle. (C) Schematic description of the first-order forward and backward firing latencies. (D) An example of cross-interval histogram of a candidate motor units pair.

After successfully decomposing the raw surface electrophysiological signals into constituent motor unit action potential trains, all the firing rate curves of the identified motor units were smoothed using a Hanning filter with a 2-s duration, and a self-developed code in the M Matlab programming language (Version 7.5.0, The Mathworks, Inc., Nattick, MA, USA) was used for further analysis. Fig 2A and 2B illustrates the individual firings and firing rates of the each identified motor units of the unilateral sternocleidomastoid muscle detected at 25% MVC during cervical isometric flexion.

### Recruitment characteristics of the motor units of the sternocleidomastoid muscles

Subsequently, three characteristic parameters were extracted to describe the recruitment electrophysiological properties of each identified motor units of the sternocleidomastoid muscles. Except for the recruitment threshold, which is defined as the relative force level (% MVC) at which the initial firing instance begins to discharge, the initial firing rate indicates the representative firing rate at which the initial firing instance occurs and the mean firing rate is defined as the average number of pulses per second (pps) during the duration of the constant force (10 s) when performing the prescribed motor task (Fig 2(B)).

### Motor unit short-term synchronization

To assess the strength of the motor unit synchronization, the short-term synchronization index of each unique possible motor unit pair was adopted as described by De Luca et al. [43, 44]. Two candidate motor unit action potential trains with recruitment threshold differences of less than 5% MVC were selected for comparison. For any candidate motor unit pair, the motor unit with fewer firing instances during the plateau period of the firing rate was taken as the reference motor unit, and the other one was designated the test motor unit. Then, a cross-interval histogram was constructed by accumulating the first-order forward and backward firing latency of the test motor unit while comparing each firing instance of the reference motor unit during the plateau period of the firing rate (Fig 2C and 2D). The width of the cross-interval histogram was set to plus-minus the mean inter-instance interval of the reference motor unit, and the binwidth resolution was set as 2 ms. The mean value and 95% confidence interval of the count of each firing latency in the cross-interval histogram were then calculated. Any accumulating firing peaks surpassing the 95% confidence interval were designated significantly synchronous firings, whereas firing peaks that fell below the 95% confidence interval were considered occasional firings. Finally, the short-term synchronization index (short-term sync index) of each motor unit pair was determined by computing the percentage of significantly synchronous firings occurring within ±6 ms of zero time latency normalized to the number of firing instances of the reference motor unit, as calculated with the following formula [33, 45]:

$$Short-term\;Sync\;Index=[\left(N_{ssf}-N_{mf}\right)/N_{tf}]\times 100\%$$

where *N*_*ssf*_ is the number of the significantly synchronous firing instances that occur within ±6 ms of zero time latency. *N*_*mf*_ denotes the mean value of the count of each firing latency in the observed cross-interval histogram. *N*_*tf*_ represents the total number of firing instances of the reference motor unit.

### Statistical analysis

Descriptive statistics were first undertaken to obtain the group mean and standard deviations (SD) for the demographic data. The relative strength of the relationships between the initial and mean firing rates and the recruitment threshold of each identified motor unit in asymptomatic individuals and in patients with MND were verified using Pearson’s correlation analysis. Moreover, the correlation between the short-term synchronization and mean recruitment threshold was assessed using grouped data with class intervals of 5% MVC for all motor unit pairs. Furthermore, a parametric independent samples *t*-test was conducted to compare the differences between the initial mean firing rates and short-term synchronization of all identified motor units in increments of 5% MVC in asymptomatic individuals and in patients with MND if the normal distribution of dependent variables was verified; otherwise, a non-parametric Mann–Whitney *U* test was applied. Significance was set at *P* < 0.05 for all statistical analyses.

## Results

**Fig 3:**
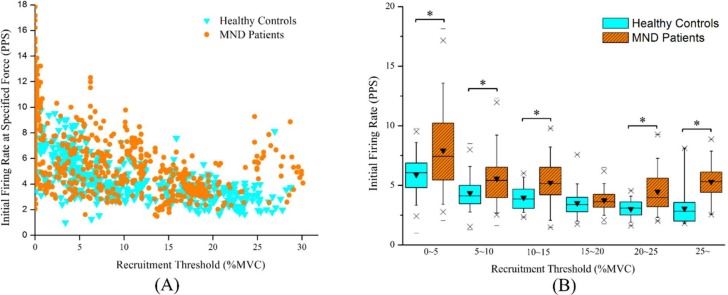
Electrophysiological characteristics of motor units at initial recruitment. (A) The initial firing rates at recruitment plotted as functions of the recruitment threshold during executing 25% MVC isometric flexion and (B) the boxplot for comparisons of the initial firing rate in increments of 5% MVC between patients with MND and asymptomatic individuals (*p<0.05).

**Fig 4:**
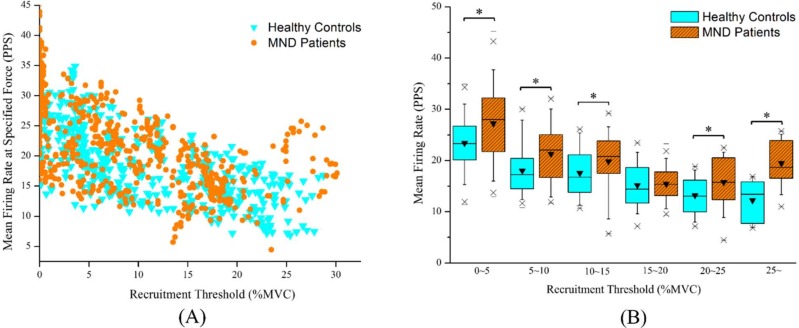
Electrophysiological characteristics of motor units during isometric flexion. (A) The mean firing rates at recruitment plotted as functions of the recruitment threshold during executing 25% MVC isometric flexion and (B) the boxplot for comparisons of the mean firing rate in increments of 5% MVC between patients with MND and asymptomatic individuals (*p<0.05).

In total, 479 motor units from asymptomatic individuals and 612 motor units from patients with MND were successfully decomposed into constituent motor unit action potential trains. Undoubtedly, there were stronger inverse relationships between the initial, mean firing rates and the recruitment thresholds for the sternocleidomastoid muscles in asymptomatic individuals (r = -0.633, *P* < 0.05 and r = -0.652, *P* < 0.05) and in patients with MND (r = -0.524, *P* < 0.05 and r = -0.617, *P* < 0.05). Figs 3 and 4 depict the distributions of the initial and mean firing rates in increments of 5% MVC for the asymptomatic individuals and the patients with MND, respectively. The results revealed that the identified motor units of the sternocleidomastoid muscles displayed significantly higher initial and mean firing rates in patients with MND than in asymptomatic individuals (*P* < 0.05) except for those exhibiting motor units with recruitment thresholds corresponding to 15~20% MVC. The detailed motor unit firing behavior of the sternocleidomastoid muscles for the asymptomatic individuals and the patients with MND is summarized in the Table 2.

**Table 2:** Detailed motor unit firing behavior of the sternocleidomastoid muscles for patients with MND and asymptomatic individuals (unit: pps (pulses per second)).

	Patients with MND		Asymptomatic Individuals	
Mean recruitment threshold	Initial firing rate	Mean firing rate	Initial firing rate	Mean firing rate
**0~5% MVC**	7.93 (3.28)*	27.24 (6.82)*	5.90 (1.55)	23.39 (4.62)
**5~10% MVC**	5.57 (2.15)*	21.19 (5.42)*	4.36 (1.25)	18.00 (4.62)
**10~15% MVC**	5.23 (1.83)*	19.80 (5.46)*	3.98 (0.97)	17.52 (4.37)
**15~20% MVC**	3.75 (0.85)	15.43 (3.07)	3.52 (1.03)	15.16 (3.98)
**20~25% MVC**	4.48 (1.76)*	15.75 (4.96)*	3.03 (0.69)	13.19 (3.42)
**25~ % MVC**	5.30 (1.54)*	19.47 (4.37)*	3.06 (1.49)	12.21 (4.04)

*Significantly higher value in patients with MND than in asymptomatic individuals (*P* < 0.05)

**Fig 5:**
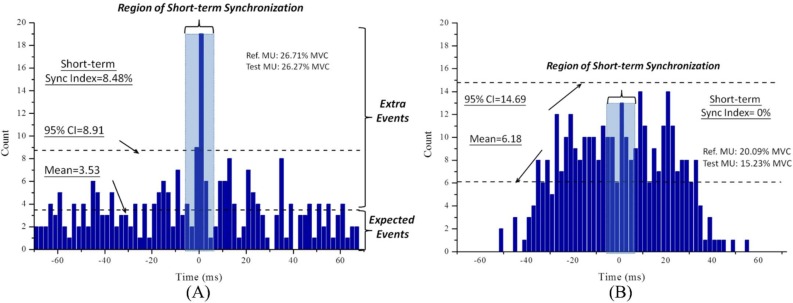
Schematic representations of cross-interval histograms of short-term synchronization. Examples of cross-interval histograms from activated motor units pair with (A) and without (B) short-term synchronization.

**Fig 6:**
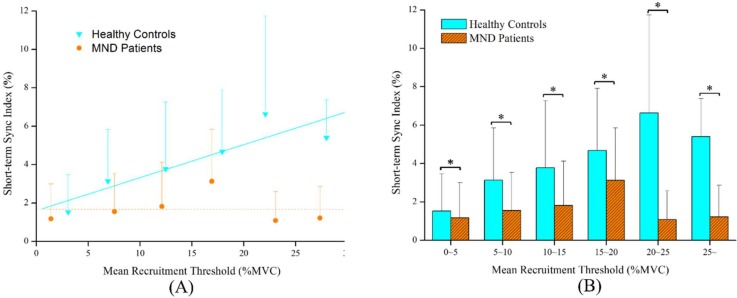
Characteristics of short-term synchronization of all identified motor units pairs. (A) The grouped linear regression analysis between short-term synchronization and mean recruitment threshold for all identified motor units pairs of the sternocleidomastoid muscles in increments of 5% MVC and (B) the histogram for comparisons of the short-term synchronization in increments of 5% MVC between patients with MND and asymptomatic individuals (*p<0.05) (for asymptomatic individuals: R2 = 0.811, p<0.01, Sync Index = 0.17*Threshold+1.60, for patients with MND: R2 = 0.058, p = 0.65, Sync Index = 0.22*Threshold+1.67).

Moreover, the degrees of short-term synchronization of all possible motor unit pairs from the sternocleidomastoid muscles were determined to characterize the neuromuscular control strategies in asymptomatic individuals and in patients with MND. Fig 5 provides schematic representations of cross-interval histograms from the occurrence and absence of short-term synchronization for two identified motor unit pairs. In the current work, a total of 2,193 motor unit pairs from asymptomatic individuals and 2,976 motor unit pairs from patients with MND were computed. Fig 6 shows the grouped linear regression analysis of the short-term synchronization and the mean recruitment threshold for all the identified motor unit pairs of the sternocleidomastoid muscles in increments of 5% MVC. The findings indicated a significant positive relationship in the asymptomatic individuals but not in the patients with MND. Additionally, the patients with MND displayed substantially lower motor unit short-term synchronization values in the sternocleidomastoid muscles compared with the asymptomatic individuals.

## Discussion

Existing evidence has substantiated the hierarchical control of motor units during voluntary isometric contraction in various neuromuscular systems, such as the vastus lateralis, vastus medialis, first dorsal interosseous and tibialis anterior muscles [43, 46–48], indicating that earlier-recruited, lowed-threshold motor units possess higher firing rates than later-recruited, higher-threshold ones. This “onion skin” phenomenon is also observed in the sternocleidomastoid muscles of asymptomatic individuals and even in patients with MND. Additionally, the novel findings of the current work showed significantly higher initial and mean motor unit firing rates in patients with MND than in asymptomatic individuals during the isometric flexion of the sternocleidomastoid muscles. The patients with MND also had reduced motor unit short-term synchronization of the sternocleidomastoid muscles. In summary, the observations obtained in this study affirm the use of altered neuromuscular control strategies in neck-related muscles among patients with neck pain in contrast with asymptomatic individuals.

When considering the electrophysiological properties of motor units, the higher values of initial and mean firing rates in the patients with MND shown here were not identical to the findings of Falla et al., who reported reduced modulation of the discharge rates of the identified motor units in the sternocleidomastoid muscles of women with chronic neck pain [49]. The contradictory observations are mainly attributable to different experimental methodologies. In the current work, the participants conducted isometric contraction at 25% MVC according to their personal muscle contraction capabilities, whereas the subjects in the investigation of Falla et al. performed isometric contraction at a constant force of 15N [49]. Different testing positions of the neck (sitting and lying [either prone or supine]), moreover, could substantially modulate different strategies for recruiting the motor units of the cervical paraspinal muscles during force production [50]. These major differences in experimental manipulations would result in opposite findings.

Another important issue should be worthy of discussing. As we know, the excitability of the motoneurone was gradually suppressed during sub-maximal fatiguing contractions [51] and the decrease of motor unit firing rate was correlated to intensity of muscle pain [52]. However, all patients from our experimental group were rated as having mild disability and no one claimed any trigger points on the sternocleidomastoid muscles while volunteer enrollment. In addition, no participants complained any occurrences of discomfort or painful condition on the sternocleidomastoid muscles during and after experiment. That is, the musculoskeletal physiological phenomena observed from the experimental participants of the current investigation supported the argument that increased activation of the superficial cervical flexor muscles is a neuromuscular control strategy to compensate for the abnormal activation of the deep cervical flexor muscles in patients with neck pain [4, 5]. This compensatory neuromuscular strategy might display distinct modulations of neuromuscular electrophysiological properties compared with painful muscular condition. Since the recruitment of the motor units or the pattern of the discharge rates of activated motor units are the two major elements for modulating mechanical output of neuromuscular systems [18], manipulations of recruiting more motor units or reinforcing discharge rates of activated motor units from uninjured muscles (superficial cervical flexor muscles) would make it possible to have comparable functions to complete prescribed motor task. Consequently, our current findings could rationally deduce reinforcement of discharge rates of activated motor units in the sternocleidomastoid muscles at least would be one of the neuromuscular adaptions to compensate for the abnormal activation of the deep cervical flexor muscles in patients with MND. This also would be why the hyper-activations of the superficial cervical flexor muscles are commonly identified for patients with neck pain [15, 16, 53]. Secondly, γ-muscle spindle afferent-mediated muscle stiffness [54] is a common clinical symptom in the superficial cervical muscles for patients with neck pain [55]. The discharge rates of activated motor units were likely to be augmented to overcome muscle stiffness of the sternocleidomastoid muscles to successfully achieve prescribed motor task in patients with MND. Finally, another candidate possibility would be that a significantly predominant percentage of fast-twitch type II muscle fibers requires greater simulation rates to accomplish twitch fusion for patients with neck pain [56, 57].

Regarding motor unit short-term synchronization, significant dependence of short-term synchronization on the mean recruitment threshold in each identified motor unit pair was documented in asymptomatic individuals. These results agree with previous observations of stronger short-term synchronization of the motor unit pairs with high-mean recruitment thresholds than with low-mean recruitment thresholds [33, 45, 58]. It is conceivable that motor unit short-term synchronization serves as a deliberate strategy for neuromuscular activation by attempting to produce efficient mechanical output of the neuromuscular systems [45]. Unquestionably, the results here showed statistically lower short-term synchronization of the motor unit pairs in patients with MND. Distinct modulations of motor unit synchronization among patients with MND and asymptomatic individuals observed in the present investigation subscribe to the assertion that patients with MND used altered neuromuscular control strategies to achieve the prescribed task compared to asymptomatic subjects. Since the plastic modulation of motor unit synchronization is generally thought to originate from common synaptic input branched presynaptic neurons [31, 32], it is most likely that the reduction in motor unit synchronization reflects the poor cortico-motoneuronal connections, which would be consistent with decreases in muscular strength and presences of jerky or irregular movements for patients with neck pain [2, 9, 10]. Furthermore, another interesting issue should be further elaborated. The findings of the present investigation found patients with MND demonstrated the elevated initial and mean firing rates of the sternocleidomastoid muscles but displayed substantially lower motor unit synchronization values. Consequently, these observations would imply a trend towards reduced neuromuscular efficiency in the sternocleidomastoid muscles during executing the required motor task in subjects with neck pain [53].

Finally, some experimental limitations of the current work should be mentioned. Firstly, the sternocleidomastoid and anterior scalene muscles actually are major superficial cervical muscles and are believed to contribute for the voluntary cervical flexion [59]. Theoretically, simultaneous observations of myoelectric manifestations of these two muscles would clarify more detailed relationships between fundamental electrophysiological properties and neuromuscular controls for patient with neck pain. Unfortunately, the system for acquiring electromyography signals adopted in the current work just could provide only two acquiring array sensors until now due to substantial technical limitations. Considering the technical restrictions and the functional significances of these two superficial cervical muscles, only the surface electrophysiological signals from the bilateral sternocleidomastoid muscles were identified. Secondly, a relative low intensity (25% MVC) and short duration of isometric contraction rather than a higher level and long duration of force was used for further comparison in this work. Despite finding greater fatigability of the sternocleidomastoid muscles in patients with neck pain, this experimental manipulation failed to explore the fatigue effects of fundamental electrophysiological properties on the motor units of the sternocleidomastoid muscles. However, a low level of muscular contraction (25% MVC) is commonly regarded as a useful and objective myoelectric condition for exploring the physiological properties of neuromuscular dysfunction in patients with neck pain [53], and it is well known that activities of daily living place low biomechanical demands on the sternocleidomastoid muscles. That is, a low level of muscular contraction seems to authentically reflect the electrophysiological nature of the sternocleidomastoid muscles. Sampling error is another limitation of this study, as there was a predominance of females in the sample. The experimental participants may not be representative of the general population. For these reasons, the present findings should be interpreted with caution.

In summary, the present study showed that the identified motor units of the sternocleidomastoid muscles displayed significantly higher initial and mean firing rates in patients with MND than in asymptomatic individuals, except for the identified motor units with recruitment thresholds corresponding to 15~20% MVC. Simultaneously, the patients with MND displayed lower motor unit short-term synchronization values of the sternocleidomastoid muscles compared with asymptomatic individuals. These convincing findings suggest that patients with MND used altered neuromuscular control strategies, such as the reinforcement of motor unit recruitment firing rates in the sternocleidomastoid muscles, to achieve the target force while performing cervical isometric flexion. Furthermore, the motor units of the MND patients also exhibited less efficient neural recruitment strategies than asymptomatic individuals when executing the required motor tasks.

## References

[1] Borghouts JA, Koes BW, Bouter LM. The clinical course and prognostic factors of non-specific neck pain: a systematic review. Pain. 1998;77: 1–13. 9755013 10.1016/S0304-3959(98)00058-X

[2] Chiu TT, Sing KL. Evaluation of cervical range of motion and isometric neck muscle strength: reliability and validity. Clin Rehabil. 2002;16: 851–858. 12501947 10.1191/0269215502cr550oa

[3] Guo LY, Lee SY, Lin CF, Yang CH, Hou YY, Wu WL, et al Three-dimensional characteristics of neck movements in subjects with mechanical neck disorder. J Back Musculoskelet Rehabil. 2012;25: 47–53. 10.3233/BMR-2012-0309 22398266

[4] Hagen KB, Harms-Ringdahl K, Enger NO, Hedenstad R, Morten H. Relationship between subjective neck disorders and cervical spine mobility and motion-related pain in male machine operators. Spine (Phila Pa 1976). 1997;22: 1501–1507. 9231970 10.1097/00007632-199707010-00015

[5] Hanten WP, Olson SL, Russell JL, Lucio RM, Campbell AH. Total head excursion and resting head posture: normal and patient comparisons. Arch Phys Med Rehabil. 2000;81: 62–66. 10638878 10.1016/s0003-9993(00)90223-5

[6] Johnston V, Jull G, Souvlis T, Jimmieson NL. Neck movement and muscle activity characteristics in female office workers with neck pain. Spine (Phila Pa 1976). 2008;33: 555–563. 18317202 10.1097/BRS.0b013e3181657d0d

[7] Jordan A, Mehlsen J, Ostergaard K. A comparison of physical characteristics between patients seeking treatment for neck pain and age-matched healthy people. J Manipulative Physiol Ther. 1997;20: 468–475. 9310902

[8] Lee H, Nicholson LL, Adams RD. Cervical range of motion associations with subclinical neck pain. Spine (Phila Pa 1976). 2004;29: 33–40. 14699273 10.1097/01.BRS.0000103944.10408.BA

[9] Yang CC, Su FC, Guo LY. A new concept for quantifying the complicated kinematics of the cervical spine and its application in evaluating the impairment of clients with mechanical neck disorders. Sensors (Basel). 2012;12: 17463–17475. 23247412 10.3390/s121217463 PMC3571848

[10] Yang CC, Su FC, Guo LY. Comparison of neck movement smoothness between patients with mechanical neck disorder and healthy volunteers using the spectral entropy method. Eur Spine J. 2014;23: 1743–1748. 10.1007/s00586-014-3413-9 24943642

[11] Bernard BE. Musculoskeletal disorders and workplace factors: a critical review of epidemiologic evidence for work-related musculoskeletal disorders of the neck, upper extremity, and low back Cincinnati, OH: U.S. Department of Health and Human Services, Centers for Disease Control and Prevention, National Institute for Occupational Safety and Health, DHHS (NIOSH) 1997.

[12] Korhonen T, Ketola R, Toivonen R, Luukkonen R, Hakkanen M, Viikari-Juntura E. Work related and individual predictors for incident neck pain among office employees working with video display units. Occup Environ Med. 2003;60: 475–482. 10.1136/oem.60.7.475 12819280 PMC1740578

[13] Palmer KT, Smedley J. Work relatedness of chronic neck pain with physical findings—a systematic review. Scand J Work Environ Health. 2007;33: 165–191. 17572827 10.5271/sjweh.1134

[14] Ariens GA, van Mechelen W, Bongers PM, Bouter LM, van der Wal G. Psychosocial risk factors for neck pain: a systematic review. Am J Ind Med. 2001;39: 180–193. 11170160 10.1002/1097-0274(200102)39:2<180::aid-ajim1005>3.0.co;2-#

[15] Falla D, Bilenkij G, Jull G. Patients with chronic neck pain demonstrate altered patterns of muscle activation during performance of a functional upper limb task. Spine (Phila Pa 1976). 2004;29: 1436–1440. 15223935 10.1097/01.brs.0000128759.02487.bf

[16] Falla D, Rainoldi A, Merletti R, Jull G. Myoelectric manifestations of sternocleidomastoid and anterior scalene muscle fatigue in chronic neck pain patients. Clin Neurophysiol. 2003; 114: 488–495. 12705429 10.1016/s1388-2457(02)00418-2

[17] Falla DL, Jull GA, Hodges PW. Patients with neck pain demonstrate reduced electromyographic activity of the deep cervical flexor muscles during performance of the craniocervical flexion test. Spine (Phila Pa 1976). 2004;29: 2108–2114. 15454700 10.1097/01.brs.0000141170.89317.0e

[18] Binder MD, Mendell LM. The Segmental motor system New York: Oxford University Press; 1990.

[19] Falla D, Farina D. Non-uniform adaptation of motor unit discharge rates during sustained static contraction of the upper trapezius muscle. Exp Brain Res. 2008;191: 363–370. 10.1007/s00221-008-1530-6 18704381

[20] Gazzoni M, Farina D, Merletti R. A new method for the extraction and classification of single motor unit action potentials from surface EMG signals. J Neurosci Methods. 2004;136: 165–177. 10.1016/j.jneumeth.2004.01.002 15183268

[21] Holtermann A, Roeleveld K, Karlsson JS. Inhomogeneities in muscle activation reveal motor unit recruitment. J Electromyogr Kinesiol. 2005;15: 131–137. 10.1016/j.jelekin.2004.09.003 15664143

[22] Kallenberg LA, Hermens HJ. Motor unit action potential rate and motor unit action potential shape properties in subjects with work-related chronic pain. Eur J Appl Physiol. 2006;96: 203–208. 10.1007/s00421-004-1215-1 15455237

[23] Kallenberg LA, Hermens HJ. Motor unit properties of biceps brachii during dynamic contractions in chronic stroke patients. Muscle Nerve. 2011;43: 112–119. 10.1002/mus.21803 20928907

[24] Kleine BU, Blok JH, Oostenveld R, Praamstra P, Stegeman DF. Magnetic stimulation-induced modulations of motor unit firings extracted from multi-channel surface EMG. Muscle Nerve. 2000;23: 1005–1015. 10882994 10.1002/1097-4598(200007)23:7<1005::aid-mus2>3.0.co;2-2

[25] Kleine BU, van Dijk JP, Lapatki BG, Zwarts MJ, Stegeman DF. Using two-dimensional spatial information in decomposition of surface EMG signals. J Electromyogr Kinesiol. 2007;17: 535–548. 10.1016/j.jelekin.2006.05.003 16904342

[26] Kleine BU, van Dijk JP, Zwarts MJ, Stegeman DF. Inter-operator agreement in decomposition of motor unit firings from high-density surface EMG. J Electromyogr Kinesiol. 2008;18: 652–661. 10.1016/j.jelekin.2007.01.010 17363274

[27] Lukacs M. Electrophysiological signs of changes in motor units after ischaemic stroke. Clin Neurophysiol. 2005;116: 1566–1570. 10.1016/j.clinph.2005.04.005 15905127

[28] Madeleine P, Leclerc F, Arendt-Nielsen L, Ravier P, Farina D. Experimental muscle pain changes the spatial distribution of upper trapezius muscle activity during sustained contraction. Clin Neurophysiol. 2006;117: 2436–2445. 10.1016/j.clinph.2006.06.753 16996301

[29] Rau G, Disselhorst-Klug C. Principles of high-spatial-resolution surface EMG (HSR-EMG): single motor unit detection and application in the diagnosis of neuromuscular disorders. J Electromyogr Kinesiol. 1997;7: 233–239. 11369266 10.1016/s1050-6411(97)00007-2

[30] Zazula D, and Holobar A. An approach to surface EMG decomposition based on higher-order cumulants. Comput Methods Programs Biomed. 2005;80 Suppl 1: S51–60. 16520144 10.1016/s0169-2607(05)80006-9

[31] Kirkwood PA, Sears TA, Tuck DL, Westgaard RH. Variations in the time course of the synchronization of intercostal motoneurones in the cat. J Physiol, 1982 327: p. 105–35. 7120134 10.1113/jphysiol.1982.sp014223 PMC1225100

[32] Sears T.A. and Stagg D., Short-term synchronization of intercostal motoneurone activity. J Physiol, 1976 263(3): p. 357–81. 1018273 10.1113/jphysiol.1976.sp011635 PMC1307707

[33] Datta AK, Stephens JA. Synchronization of motor unit activity during voluntary contraction in man. J Physiol. 1990;422: 397–419. 2352185 10.1113/jphysiol.1990.sp017991 PMC1190139

[34] De Luca CJ, Roy AM, and Erim Z. Synchronization of motor-unit firings in several human muscles. Neurophysiol. 1993;70: 2010–2023. 10.1152/jn.1993.70.5.2010 8294967

[35] Semmler JG. Motor unit synchronization and neuromuscular performance. Sport Sci Rev. 2002;30: 8–14. 10.1097/00003677-200201000-00003 11800501

[36] Schmied A, Pouget J, Vedel JP. Electromechanical coupling and synchronous firing of single wrist extensor motor units in sporadic amyotrophic lateral sclerosis. Clin Neurophysiol. 1999;110: 960–974. 10400212 10.1016/s1388-2457(99)00032-2

[37] Farmer SF, Ingram DA, Stephens JA. Mirror movements studied in a patient with Klippel-Feil syndrome. J Physiol. 1990;428: 467–484. 2231422 10.1113/jphysiol.1990.sp018222 PMC1181657

[38] Datta AK, Farmer SF, Stephens JA. Central nervous pathways underlying synchronization of human motor unit firing studied during voluntary contractions. J Physiol. 1991;432: 401–425. 1886061 10.1113/jphysiol.1991.sp018391 PMC1181332

[39] Farmer SF, Swash M, Ingram DA, Stephens JA. Changes in motor unit synchronization following central nervous lesions in man. J Physiol. 1993;463: 83–105. 8246205 10.1113/jphysiol.1993.sp019585 PMC1175334

[40] Gibbs J, Harrison LM, Stephens JA, Evans AL. Does abnormal branching of inputs to motor neurones explain abnormal muscle cocontraction in cerebral palsy? Dev Med Child Neurol. 1999;41: 465–472. 10454230

[41] Rose J, McGill KC. Neuromuscular activation and motor-unit firing characteristics in cerebral palsy. Dev Med Child Neurol. 2005;47: 329–336. 15892375 10.1017/s0012162205000629

[42] Vernon H, Mior S. The Neck Disability Index: a study of reliability and validity. J Manipulative Physiol Ther. 1991;14: 409–415. 1834753

[43] De Luca CJ, Hostage EC. Relationship between firing rate and recruitment threshold of motoneurons in voluntary isometric contractions. J Neurophysiol. 2010;104: 1034–1046. 10.1152/jn.01018.2009 20554838 PMC2934917

[44] Nawab SH, Chang SS, De Luca CJ. High-yield decomposition of surface EMG signals. Clin Neurophysiol. 2010;121: 1602–1615. 10.1016/j.clinph.2009.11.092 20430694 PMC2932793

[45] Defreitas JM, Beck TW, Ye X, Stock MS. Synchronization of low- and high-threshold motor units. Muscle Nerve. 2014;49: 575–583. 10.1002/mus.23978 23893653

[46] De Luca CJ, Contessa P. Hierarchical control of motor units in voluntary contractions. Neurophysiol. 2012;107: 178–195. 10.1152/jn.00961.2010 PMC3349682 21975447

[47] De Luca CJ, Erim Z. Common drive of motor units in regulation of muscle force. Trends Neurosci. 1994;17: 299–305. 7524216 10.1016/0166-2236(94)90064-7

[48] Stock MS, Beck TW, Defreitas JM. Effects of fatigue on motor unit firing rate versus recruitment threshold relationships. Muscle Nerve. 2012;45: 100–109. 10.1002/mus.22266 22190315

[49] Falla D, Lindstrøm R, Rechter L, Farina D. Effect of pain on the modulation in discharge rate of sternocleidomastoid motor units with force direction. Clin Neurophysiol. 2010;121: 744–753. 10.1016/j.clinph.2009.12.029 20097603

[50] Gogia PP, Sabbahi MA. Changes in Fatigue Characteristics of Cervical Paraspinal Muscles with Posture. Spine (Phila Pa 1976). 1991;16: 1135–1140. 1754932 10.1097/00007632-199110000-00001

[51] McNeil CJ, Giesebrecht S, Gandevia SC, Taylor JL. Behaviour of the motoneurone pool in a fatiguing submaximal contraction. J Physiol. 2011;589: 3533–3544. 10.1113/jphysiol.2011.207191 21606110 PMC3167116

[52] Farina D, Arendt-Nielsen L, Merletti R, Graven-Nielsen T. Effect of Experimental Muscle Pain on Motor Unit Firing Rate and Conduction Velocity. J Neurophysiol. 2004;91: 1250–1259. 10.1152/jn.00620.2003 14614105

[53] Falla D, Jull G, Edwards S, Koh K, Rainoldi A. Neuromuscular efficiency of the sternocleidomastoid and anterior scalene muscles in patients with chronic neck pain. Disabil Rehabil. 2004;26: 712–717. 10.1080/09638280410001704287 15204493

[54] Sjolander P, Johansson H, Djupsjobacka M. Spinal and supraspinal effects of activity in ligament afferents. J Electromyogr Kinesiol. 2002;12: 167–176. 12086810 10.1016/s1050-6411(02)00017-2

[55] Fejer R, Kyvik KO, Hartvigsen J. The prevalence of neck pain in the world population: a systematic critical review of the literature. Eur Spine J. 2006;15: 834–848. 10.1007/s00586-004-0864-4 15999284 PMC3489448

[56] Uhlig Y, Weber BR, Grob D, Müntener M. Fiber composition and fiber transformations in neck muscles of patients with dysfunction of the cervical spine. J Orthop Res. 1995;13: 240–249. 10.1002/jor.1100130212 7722761

[57] Bellemare F, Woods JJ, Johansson R, Bigland-Ritchie B. Motor-unit discharge rates in maximal voluntary contractions of three human muscles. J Neurophysiol. 1983;50: 1380–1392. 6663333 10.1152/jn.1983.50.6.1380

[58] Schmied A, Vedel JP, Pagni S. Human spinal lateralization assessed from motoneurone synchronization: dependence on handedness and motor unit type. J Physiol. 1994:480; 369–387. 7869252 10.1113/jphysiol.1994.sp020367 PMC1155853

[59] Standring S. Gray's anatomy: the anatomical basis of clinical practice 41^st^ ed. New York: Elsevier Limited; 2016.

